# Cholesterol Synthesis Increased in the MMI-Induced Subclinical Hypothyroidism Mice Model

**DOI:** 10.1155/2017/7921071

**Published:** 2017-03-12

**Authors:** Yongfeng Song, Xiujuan Zhang, Wenbin Chen, Ling Gao

**Affiliations:** ^1^Department of Endocrinology and Metabolism, Shandong Provincial Hospital Affiliated to Shandong University, Jinan, Shandong 250021, China; ^2^Institute of Endocrinology and Metabolism, Shandong Academy of Clinical Medicine, Jinan, Shandong 250021, China; ^3^Scientific Center, Shandong Provincial Hospital Affiliated to Shandong University, Jinan, Shandong 250021, China

## Abstract

Subclinical hypothyroidism (SCH) is defined as increased serum thyroid-stimulating hormone (TSH) concentrations and normal serum thyroid hormone (TH) levels as well as an increased serum cholesterol level, which is an important cause of secondary hypercholesterolemia and cardiovascular diseases. Some studies have demonstrated a direct effect of TSH on cholesterol metabolism via in vivo and in vitro experiments. However, because no suitable SCH model has been established until now, the changes in cholesterol synthesis that occur in SCH patients remain unknown. Here, we establish an SCH mouse model by using long-term low-dose MMI administered in drinking water. Compared with the control group, the MMI-treated mice had elevated circulating TSH levels, but the serum FT3 levels in these mice did not change. Additionally, the TC levels increased in both the serum and liver of the experimental mice. Both the protein expression and activity of hepatic HMGCR, the rate-limiting enzyme for cholesterol synthesis in the liver, increased in these mice. We also found that the SCH mice had decreased phospho-HMGCR and phospho-AMPK expression, while the expression of AMPK showed no change. In conclusion, we established a suitable SCH model in which cholesterol synthesis is increased.

## 1. Introduction

Hypothyroidism is a clinical syndrome involving thyroid hormone (TH) deficiency and elevated serum total cholesterol (TC), which can result in hypercholesterolemia [1, 2]. The underlying mechanism has widely been thought to be TH deficiency. However, in patients with subclinical hypothyroidism, TH levels stay within normal range, and only thyroid-stimulating hormone (TSH) increases; these patients also have elevated serum TC levels [1, 3, 4]. These observations raise the question of whether increased TSH is also associated with elevated cholesterol. Several clinical studies in recent years addressed this issue and showed a correlation in hypothyroidism between high serum cholesterol and high TSH levels, the latter of which is typically used to indicate the severity of the hypothyroidism [5–7].

HMG-CoA reductase (HMGCR), which catalyzes the conversion of HMG-CoA to mevalonate, is the rate-limiting enzyme in cholesterol biosynthesis. The liver plays a major role in regulating cholesterol levels, so hepatic HMGCR is responsible for the majority of the regulated cholesterol synthesis in the body. HMGCR is a highly regulated enzyme in terms of protein amount and activation state. Control is exerted by the synthesis and degradation rates of the enzyme and by posttranslational processes such as phosphorylation or dephosphorylation. HMGCR is physiologically present in the cell in both the unphosphorylated active form (30%) and the phosphorylated inactive form (70%). The ratio of unphosphorylated to total HMGCR indicates the activation state of the enzyme. This is based on the phosphorylation state of serine 871, which resides in the catalytic domain. Specific kinases (mainly AMP-dependent kinase, AMPK) [8] are responsible for the interconversion of phosphorylated and unphosphorylated HMGCR.

In our previous studies, we identified that TSHR was expressed in hepatocytes and that TSH could increase the production of cAMP via TSHR, suggesting TSH may, to some extent, play a physiopathological role in the liver in addition to its role in regulating the thyroid via TSHR. We have also demonstrated that TSH can increase the expression of HMGCR by upregulating the cAMP/PKA/CREB signaling pathway and can decrease its phosphorylation via AMP-activated protein kinase (AMPK) in the liver [9, 10]. However, compelling evidence for these activities in animals is still lacking, and the role of cholesterol synthesis in the SCH model is still unclear.

In the present study, we established an SCH mouse model using long-term low-dose MMI in drinking water, and changes in the expression of HMGCR and in the phosphorylation states of AMPK and HMGCR were explored.

## 2. Methods

### 2.1. Animal Experiments

Male Kunming mice (aged 6–8 weeks) were obtained from the Laboratory Animal Center of Shandong University. The animals were fed with laboratory chow and water ad libitum. The mice were housed at 23°C in a 12 h light-dark cycle with a humidity-controlled (60%) environment. All procedures were carried out with the approval of the Institutional Animal Care and complied with the *Guide for the Care and Use of Laboratory Animals*. After one week for acclimation, the mice were divided into two groups: the SCH group (*n* = 13) was given 4 mg/kg·d methimazole (MMI, Xing-Jian-Fang-Tian Company Ltd. Peking, China) in their drinking water; the control group (*n* = 12) was given an equal volume of untreated water. The volume of drinking water for each mouse was tested every three days, and the individual dose of MMI was adjusted based on body weight changes during the experimental process. At the end of the second month of MMI treatment, all mice were sacrificed. The serum and livers of these mice were harvested for assay. The serum samples were stored at −80°C, and liver samples were stored in liquid nitrogen. Serum thyroid hormone (FT3 and FT4) levels and TSH levels were determined with a competition radioimmunoassay (RIA) binding assay kit (Jiuding, Tianjin, China) following the manufacturer's instructions.

### 2.2. Western Blot Assay

Equal amounts (80 *μ*g) of protein samples were resolved by 10% SDS-PAGE and then electrotransferred to nitrocellulose membranes (Millipore). All membranes were incubated overnight at 4°C with anti-P-HMGCR (1 : 1000, Millipore), anti-HMGCR (1 : 1000, Upstate), anti-P-AMPK (1 : 2000, CST), anti-AMPK (1 : 1000, CST), and anti-*β*-actin (1 : 10000, Abcam). After incubation with secondary antibody (Zsbio, Ltd., China), immune complexes were detected using the enhanced chemiluminescence plus detection system (Amersham). Immunoreactive bands were quantified using the Alphaimager 2200. The relative target protein levels were normalized to *β*-actin.

### 2.3. Liver HMGCR Activity Assay

The liver microsomes to be used in the assay for HMGCR activity were prepared as described by Honda et al. [11]. Briefly, 50 mg liver tissues were homogenized with a loose-fitting Teflon pestle in 4 volumes of 3 mM Tris-HCl buffer (pH 7.4) containing 0.25 M sucrose and 0.1 mM ethylenediaminetetraacetic acid (EDTA). The homogenates were centrifuged by differential ultracentrifugation, and the pellet (microsomal fraction) was suspended in storage buffer containing 100 mM potassium phosphate buffer ((pH 7.4), 1 mM EDTA, 5 mM dithiothreitol, 50 mM KCl, and 20% glycerol (v/v)). Measurement of HMGCR activity by LC-P-ESI-MS/MS (API 4000, Applied Biosystems/MDS Sciex) was carried out as described previously [11]. Briefly, the microsomes (100 *μ*g of protein) were incubated for 30 min at 37°C in a total volume of 150 *μ*L including 100 mM potassium phosphate buffer (pH 7.4) containing an NADPH generating system and 30 nmol HMG-CoA. The reaction was terminated by the addition of 20 *μ*L of 6N HCl, and the tube was allowed to stand at room temperature for 10 min to ensure lactonization of the biosynthetic MVA at pH < 1. After the addition of 10 ng [2H]MVL, MVL was extracted using diethyl ether; the ether phase supernatant was collected and evaporated to dryness under nitrogen, and then 100 *μ*L volume of toluene and 20 *μ*L of 1-(2-aminoethyl) pyrrolidine were added to the residue of the ether extract and incubated at 55°C for 60 min. After the addition of 2 mL of toluene, the mixture was purified using the Bond Elut SI cartridge (100 mg, Varian, Harbor City, CA). After evaporation, the residue was redissolved in 100 *μ*L of methanol-water (5 : 95, v/v) containing 0.1% acetic acid, and an aliquot (1 *μ*L) was injected into the LC-P-ESI-MS/MS system.

### 2.4. Liver TC Content Assay

The liver TC content was measured using a Cholesterol/Cholesteryl Ester Quantitation Kit (Biovision, California, USA) according to the manufacturer's instructions. Briefly, after the mice were anesthetized, the livers were harvested, and 10 mg of tissue was homogenized in 200 *μ*L chloroform-methanol (2 : 1, v/v). The homogenate was centrifuged for 15 min at 14,000 rpm in a microcentrifuge. The organic phase was transferred to a clean tube and vacuum-dried, and the dried lipids were redissolved in 20 *μ*L 2-propanol, 10% TritonX-100 detergent. One microliter of dissolved lipid was used per assay, and the final volume was adjusted to 50 *μ*L with Cholesterol Reaction Buffer added directly to the well(s) of a 96-well plate. Fifty microliters of Reaction Mix was added to each standard or sample well, and the plate was incubated for 1 h at 37°C away from light. The absorbance was then measured at 570 nm, and the sample TC content was calculated. Data are expressed as *μ*g cholesterol/mg wet liver weight.

### 2.5. Statistical Analysis

Data were analyzed using SPSS 17.0 software and were expressed as the means ± SDs. Statistical significance was assessed by unpaired Student's *t*-tests. A *p* value of *p* < 0.05 was considered statistically significant.

## 3. Results

### 3.1. Serum and Liver TC Levels Were Increased in SCH Mice

The characteristic feature of SCH is elevated serum TSH and normal TH levels. Compared with levels in the control group, the circulating TSH levels in the MMI-treated mice increased by 54.2% (*p* < 0.05), but neither the serum FT3 level nor the FT4 level (both *p* > 0.05) was changed in the experimental mice (Table 1), which suggested that the SCH mouse model was established successfully.

**Figure 1:**
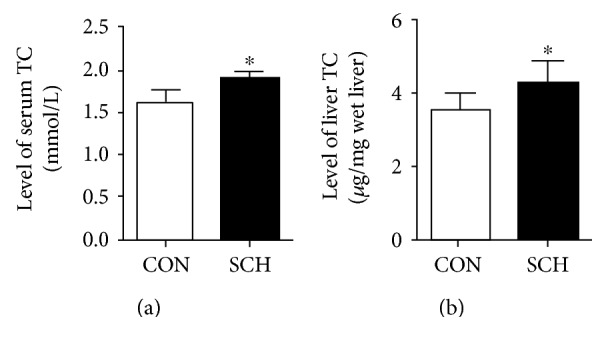
Serum and liver TC levels increased in SCH mice. Male Kunming mice were divided into two groups: mice in the SCH group were given 4 mg/kg·d methimazole in their drinking water; mice in the control group received an equal volume of distilled water. At the end of two months, the serum and livers of these mice were harvested for assay. Serum cholesterol levels (a) and liver tissue cholesterol contents (b) were assayed using a Cholesterol/Cholesteryl Ester Quantitation Kit. Data are representative of three independent experiments. ^∗^*p* < 0.05 versus control mice.

As shown in Figures 1(a) and 1(b), cholesterol levels in the SCH mice increased by 17.5% in the serum (*p* < 0.05) and by 19.1% in the liver (*p* < 0.05) compared to corresponding levels in control mice.

### 3.2. The Expression and Activity of HMGCR Were Increased in the Livers of SCH Mice

**Figure 2:**
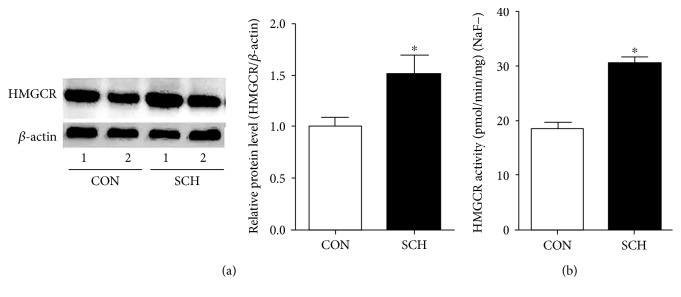
The expression and activity of HMGCR increased in the livers of SCH mice. (a) The expression of HMGCR protein was assessed using western blotting. (b) The activity of HMGCR in liver microsomes was determined by LC-P-ESI-MS/MS. Data were compiled from at least three independent experiments with triplicates in each experiment. ^∗^*p* < 0.05 versus control mice.

The expression of the hepatic HMGCR protein was 49% higher in the SCH mice than in the control group (*p* < 0.05) (Figure 2(a)). To test if the activity of HMGCR also changed in the SCH mice, we measured it using the LC-P-ESI-MS/MS system as described by Honda et al. [11]. As shown in Figure 2(b), the activity of hepatic HMGCR was elevated by 65.6% in the SCH mice relative to the control group (*p* < 0.05).

### 3.3. The Phosphorylation of HMGCR and AMPK Was Decreased in SCH Mice

**Figure 3:**
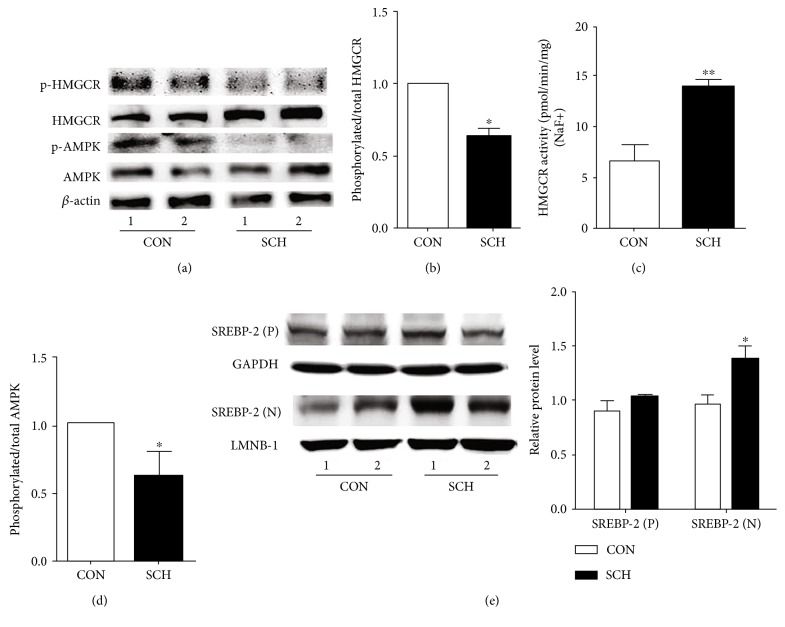
The phosphorylation of HMGCR and AMPK decreased in SCH mice. (a) The expression levels of p-HMGCR, t-HMGCR, p-AMPK, and t-AMPK in SCH and control mice were analyzed using western immunoblotting analysis. The same membranes were reprobed with anti-*β*-actin antibody to confirm equal loading of proteins for each sample. Representative results from three independent experiments are shown. (b) The ratios of p-HMGCR/HMGCR in the livers of control mice and SCH mice were assessed by semiquantitative analysis. (c) Hepatic HMGCR activity was measured in the presence of NaF. (d) The ratios of p-AMPK/AMPK in the livers of control mice and SCH mice. (e) The expression of precursor and mature SREBP-2 in SCH and control mice. Data are representative of three independent experiments. ^∗^*p* < 0.05 and ^∗∗^*p* < 0.01 versus control mice.

Because the ratio of phosphorylated to total HMGCR also serves as an indicator of the inactivation state of HMGCR, we assessed the expression of phosphorylated HMGCR and noted that, in SCH mice, the level of phosphorylated HMGCR decreased by 21% (*p* < 0.05) (Figure 3(a)), and the ratio of phosphorylated HMGCR to total HMGCR decreased by 39% (*p* < 0.05) (Figure 3(b)). To observe if the change in phosphorylated HMGCR had an effect on HMGCR activity, we also measured HMGCR activity in the presence of NaF, which preserves the phosphorylated state seen in vivo. As shown in Figure 3(c), HMGCR activity increased by 108.6% in SCH mice compared with control mice, which was a larger increase than was observed without NaF (Figure 2(b)); this result was likely due to dephosphorylation and activation of HMGCR during isolation [12, 13].

AMPK plays an important role in the regulation of HMGCR phosphorylation in the liver [10]. As shown in Figure 3(a), western blotting indicated that the expression of total AMPK did not significantly change (*p* > 0.05), whereas the expression of phosphorylated AMPK (activated) was decreased in the livers of SCH mice compared with those of the control mice (*p* < 0.05). Correspondingly, the ratio of phosphorylated AMPK to AMPK was also decreased in SCH mice compared to the control mice (*p* < 0.05, Figure 3(d)). Additionally, SREBP-2 is an important transcriptional regulator of HMGCR expression, and our previous study found that TSH can increase SREBP-2 expression in vitro [14]; we found that the expression of mature SREBP-2 increased in SCH mice compared to the control mice; however, the expression of precursor SREBP-2 had no significant change (Figure 3(e)).

## 4. Discussion

In this study, we found that MMI-treated mice exhibited elevated circulating TSH levels but unchanged serum FT3 and FT4 levels compared with control mice. Additionally, the cholesterol levels in both the serum and the livers of the SCH mice were increased. Furthermore, expression of the hepatic HMGCR protein was higher, and its activity was also increased in these mice. Finally, SCH mice had decreased p-HMGCR and p-AMPK expression compared to control mice, while AMPK expression showed no change.

As research on SCH has emerged, the use of the SCH animal model has become more common. However, to our knowledge, no suitable SCH model has been established until now. Some studies have used hemi-thyroid electrocauterization to establish an SCH phenotype, but this method is difficult to carry out [15]. In this study, we established an SCH mouse model successfully by using long-term drinking water containing a low dose of MMI. In treated mice, circulating TSH levels increased, and there were no changes in the serum FT3 levels, which fit typical SCH characteristics very well.

Consistent with our previous results that TSH can elevate cholesterol synthesis by upregulating HMGCR [9], we found that the serum and liver cholesterol levels increased, and hepatic HMGCR expression was also upregulated in SCH mice. These results further support that TSH, in addition to thyroid hormone, plays an important role in the pathogenesis of hypercholesterolemia in hypothyroidism.

Hepatic HMGCR is responsible for regulating cholesterol synthesis in the body. The equilibrium of enzyme phosphorylation-dephosphorylation plays an important role in regulating HMG-CoA reductase activity. It has been shown that dephosphorylation of HMGCR enhances its activity, whereas phosphorylation at serine 872 leads to enzyme inactivation [16]. Pallottini et al. showed that, in aged rats, HMGCR is completely dephosphorylated in the liver, which indicates full activation of the enzyme [17]; this report explained that increases in cholesterol production in the liver and higher cholesterol content in the blood occur during aging. In our study, the SCH mice showed a decrease in phosphorylated HMGCR and an increase in HMGCR activity, which was associated with an increase in cholesterol biosynthesis in the liver and serum.

AMPK is activated by increased intracellular AMP concentrations and is generally described as a “metabolite-sensing kinase.” Henin et al. indicated that an AMPK stimulator, such as AICAR, could decrease the synthesis of cholesterol in the liver [18]. Several studies reported that HMGCR can be phosphorylated in vitro by several protein kinases, such as AMPK, PKC, and Ca2+ calmodulin-dependent kinase [19, 20]. AMPK seems to be the major kinase that targets HMGCR in the liver [21]. AMPK is activated upon phosphorylation and subsequently inactivates HMG-CoA reductase via phosphorylation of the enzyme [22, 23]. In the present study, AMPK activity, which was accompanied by increased TSH levels, decreased in SCH mice. This result indicated that TSH-suppressed phosphorylated HMGCR expression may be mediated by decreasing AMPK activation.

Our study set up a novel SCH model and found that cholesterol synthesis increased in SCH mice, which provides new evidence that TSH has a direct effect on cholesterol metabolism.

## Figures and Tables

**Table 1:** Characteristics of subclinical hypothyroidism (SCH) and control mice (mean ± standard deviation).

	CON	SCH
N	10	12
LBI	0.035 ± 0.002	0.037 ± 0.003
FT3 (pmol/L)	4.09 ± 0.27	3.97 ± 0.30
FT4 (pmol/L)	2.75 ± 0.70	2.60 ± 0.10
TSH (*μ*IU/mL)	0.024 ± 0.012	0.061 ± 0.024^∗^

N: number; BW: body weight; LBI: liver weight/body weight index; FT3: free triiodothyronine; FT4: free thyroxine; and TSH: thyroid-stimulating hormone.

^∗^Indicates a significant difference of *p* < 0.05 versus control mice.
